# Discriminant analysis of ecological factors influencing sarcopenia in older people in South Korea

**DOI:** 10.3389/fpubh.2024.1346315

**Published:** 2024-05-28

**Authors:** Yoonho Ra, Ikyoung Chang, Jiyoun Kim

**Affiliations:** ^1^Institute of Human Convergence Health Science, Gachon University, Incheon, Republic of Korea; ^2^Department of Sport Coaching, Korea National Sport University, Seoul, Republic of Korea; ^3^Department of Exercise Rehabilitation, Gachon University, Incheon, Republic of Korea

**Keywords:** sarcopenia, SARC-F, ecological system, individual, microsystem, exosystem

## Abstract

This study aimed to investigate the ecological system factors that influence discrimination of sarcopenia among older individuals living in contemporary society. Data analysis included information from 618 older adults individuals aged 65 years or older residing in South Korea. To assess variations in ecological system factors related to SARC-F scores, we conducted correlation analysis and t-tests. Discriminant analysis was used to identify factors contributing to group discrimination. The key findings are summarized as follows. First, significant differences at the *p* < 0.001 level were observed between the SARC-F score groups in various aspects, including attitudes toward life, wisdom in life, health management, social support, media availability, sports environment, collectivist values, and values associated with death. Further, service environment differences were significant at *p* < 0.01 level, while social belonging and social activities exhibited significance at *p* < 0.05. Second, factors influencing group discrimination based on the SARC-F scores were ranked in the following order: health management, attitudes toward life, fear of own death, wisdom in life, physical environment, sports environment, media availability, social support, fear of the own dying, collectivist values, service environment, social activities, and social belonging. Notably, the SARC-F tool, which is used for sarcopenia discrimination, primarily concentrates on physical functioning and demonstrates relatively low sensitivity. Therefore, to enhance the precision of sarcopenia discrimination within a score-based group discrimination process, it is imperative to incorporate ecological system factors that exert a significant influence. These modifications aimed to enhance the clarity and precision of the text in an academic context.

## 1 Introduction

Advancements in modern medical technology and public health initiatives have led to an increase in life expectancy and an extension of healthy lifespans. This phenomenon has transformed the older population from a marginalized group into a significant constituent of the social fabric. This transformative process redefines old age, shifting it from a phase marked by retirement and social disengagement to one in which individuals continue to participate actively in society. As active members of society, older adults possess the rights and qualifications to be respected and enjoy the freedom granted to all members of the community (1). Consequently, this societal transformation has sparked heightened awareness of the challenges and opportunities associated with aging.

The South Korea, with a rapidly aging population on an unprecedented scale, became an aging society in 2000 and entered the aged society in 2017. As of 2023, the older population aged 65 years or older in Korea accounted for 18.4% (9,499,933) of the total population (51,558,034), and Korea is expected to become a super-aged society by 2025 (2). This means that it is the fastest-growing country in the world. This phenomenon is becoming a larger social problem as it reverses the demographic structure of the Republic of Korea with a decrease in the marriage and fertility rates.

As the demographic structure in Korea undergoes significant transformation, the well-being and role of older individuals in society have gained increasing importance. Rather than viewing them solely as individuals, the focus has shifted toward understanding and improving their quality of life as active members of society. This change recognizes the value of their contributions and seeks to enhance their overall well-being and social integration.

As the global importance of the quality of life for the older population continues the grow, discussions are unfolding concerning aging, diseases, healthcare systems, and public policies associated with the aging population phenomenon (3, 4). Numerous studies have focused on health conditions that significantly influence the quality of life in old age, particularly emphasizing diseases such as cancer and diabetes. Among these, sarcopenia (ICD-10-CM), officially recognized as a disease by the World Health Organization in 2016, has garnered global attention. Sarcopenia is characterized by a reduction in muscle strength and function, resulting in diminished muscle mass, and is linked to chronic diseases, malnutrition, and natural aging (5). Historically, the decline in muscle strength and overall muscle mass with age, accompanied by reduced physical activity, has been considered a natural consequence of the aging process, leading to social detachment in older individuals (6). However, sarcopenia is no longer considered a natural phenomenon during aging (7). This condition adversely affects an individual’s physical capabilities and has profound implications for the overall quality of life in old age, exerting a substantial impact on general health and well-being (8). This paradigm shift has transformed what was once taken for granted into a global health concern, challenging the perception of muscle loss and frailty in old age as normative aspects of human experience.

In addition, the government and local authorities actively engage in a range of initiatives aimed at maintaining economic productivity and social welfare while addressing the needs of the older population. The World Health Organization (1) emphasizes the importance of creating a societal framework with policies and environmental conditions that cater to the needs of older individuals. Such an elder-friendly community environment has been shown to have a positive impact on the well-being and lives of the older population (9, 10). The Republic of Korea’s government has underscored the significance of the environment as a fundamental human right, as enshrined in Article 35 of the Constitution. Various policies have been implemented to enhance the quality of life of older adults, including the introduction of long-term care insurance systems and the expansion of nursing facilities and hospitals (11). In alignment with global health guidelines, efforts are being made to address health issues directly related to old age. Following the WHO’s lead, South Korea introduced a diagnostic code (M62.5) for sarcopenia in 2021, officially recognizing it as a disease. This recognition has spurred various discussions and developments aimed at better understanding and management of sarcopenia and its impact on the older population.

Most studies have focused on the application of sarcopenia diagnostic tools, the prevalence rate (12–15), and the relationship between sarcopenia and other chronic diseases by collecting substantial data through measurement (16–19). Moreover, studies to identify the factors affecting sarcopenia (20, 21) have been conducted, but most of them were related to motor function or other diseases, and studies examining the influence of environmental factors are insufficient.

Bronfenbrenner (22) systematically classified and analyzed the factors affecting human developmental processes and life through ecological systems. The internal individual refers to the characteristics of the individual (e.g., sex, age, and personality). Bronfenbrenner saw the individual as an active being and that the individual develops through interaction in a social context. Microsystems refer to the activities, interpersonal relationships, and roles in which individuals experience a particular environment. The environment in the microsystem refers to a place where one can interact face-to-face, which means interaction with one’s family, peers, etc. Mesosystems are the interrelationships between two or more microsystems that actively influence an individual’s development. Exosystems do not directly affect individuals but affect events that occur within the environment. Exosystems affect microsystems and individuals and include sports facilities and public transportation in the environment. Macrosystems refer to cultures, ideologies, and institutions provided to individuals (22).

Therefore, this study aimed to investigate the ecological system factors affecting sarcopenia discrimination in the older population in South Korea. Specifically, discriminant analysis will be conducted on the ecological system factors affecting the SARC-F, which is used as a sarcopenia screening test to identify factors affecting group classification.

## 2 Materials and methods

### 2.1 Data

The study was conducted by surveying individuals aged 65 years and older residing in South Korea. The survey included a total of 100 questions covering aspects such as personal characteristics (sex, age, family structure, housing type, physical activity, etc.), sarcopenia screening questions (SARC-F), and questions related to the ecological system (e.g., ego integrity, social support, media availability, sports environment, age-friendly environment, etc.). Data were collected using face-to-face and non-face-to-face methods. A total of 762 data points were collected, and 618 data points were analyzed, excluding insincere responses. This study was approved by the Institutional Review Board of Gachon University (No. 2023–020, 22 Mar 2023).

Table 1 provides an overview of the participants’ personal characteristics. The sex distribution of the participants was 289 men (46.8%) and 329 women (53.2%), and the average age was 65.4 years. The number of participants for specific age groups was as follows: 544 (88.0%)—those in their 60s; 69 (11.2%)—those in their 70s; 5 (0.8%)—those aged 80 years and above. Regarding education level, three (0.5%) had graduated from elementary school, 19 (3.1%) completed middle school, 175 (28.3%) had graduated high school, 338 (54.7%) had graduated from university, and 83 (13.4%) had graduated school or higher. In terms of housing type, detached houses comprised 58 (9.4), apartments 452 (73.1%), multiplex housing 97 (15.7%) and other 11 (1.8). Regarding family structure, 78 (12.6%) lived in single-person households, 229 (37.1%) shared their residence with their spouses and children, 261 (42.2%) lived with only their spouses, and 50 (8.1%) were part of their children’s households.

**Table 1:** Participants’ characteristics.

	Division	Cases	Percent (%)		Division	Cases	Percent (%)
Sex	Male	289	46.8	Smoking	Yes	290	46.9
	Female	329	53.2		No	328	53.1
Age	60s	544	88.0	Drinking	Yes	422	68.3
	70s	69	11.2		No	196	31.7
	80 years and older	5	0.8				
Education	Elementary school	3	0.5	Housing type	Detached house	58	9.4
	Middle school	19	3.1		Apartment	452	73.1
	High school	175	28.3		Multiplex housing	97	15.7
	College	338	54.7				
	Graduate school or higher	83	13.4		Other	11	1.8
SARC-F	Score ≤ 3	566	91.6	Family type	Single family	78	12.6
	Score ≥ 4	52	8.4		With spouse and children	229	37.1
Physical activity	Yes	537	86.9		With spouse	261	42.2
	No	81	13.1		With children	50	8.1

### 2.2 Variables

Table 2 presents the variables used in this study. The researcher’s personal characteristics comprised eight questions on sex, age, education, smoking, drinking, family type, housing type, and periodic physical activity. The Sarcopenia Screening Question (SARC-F), developed by Malmstrom and Morley (23), comprises five questions. The questions are: (a) How much difficulty do you have in lifting and carrying 10 lb.?; (b) How much difficulty do you have walking across a room?; (c) How much difficulty do you have transferring yourself from a chair or bed?; (d) How much difficulty do you have climbing a flight of 10 stairs?; (e) How many times have you fallen in the past year? Each question focuses on different aspects of physical ability and mobility. A sarcopenia screening question was used at the most basic stage to determine sarcopenia.

**Table 2:** Description of variables.

Division	Variable name	Note
Personal characteristics	Sex, age, education, smoking, drinking, family type, housing type, physical activity	Write and Likert
Sarcopenia screening	SARC-F	Three-point Likert
Ecological system factor	Ego-integrity, goal content for exercise, social support, social relationship, media availability, sports environment, age-friendly environment, fear of death scale, collectivism	Five-point Likert

There were 115 questions related to ecological system factors, including ego integrity, goal content for exercise, social support, media availability, social relationships, sports environment, age-friendly environment, fear of death scale, and collectivism. Among the ecological system factors, the scale developed by Kim (24) was modified and supplemented according to the conceptual framework of Miller (25) and Erikson (26) to suit the purpose of the study, and the sense of self-integration was derived from five items of attitude toward life (3.082) and four items of wise life (2.223), a total of two factors and nine items, and the scale was found to be reliable in terms of attitude toward life (*α* = 0.841) and wise life (*α* = 0.701). The goal content for the exercise scale was modified and supplemented to the scale developed by Sebire et al. (27) to suit the purpose of the study. The goal content for exercise was derived from four items of social cognition (3.468), four items of social belonging (3.108), four items of health care (2.913), and three items of skill development (2.457), totaling 15 items of four factors. The reliability of this was at a good level in terms of social recognition (*α* = 0.941), social affiliation (*α* = 0.902), health management (*α* = 0.871), and skill development (*α* = 0.897).

The Social Support Scale was developed by Sallis et al. (28) and modified and supplemented by Choi (29). For social support (3.936), one factor and six items were derived. The reliability of this was *α* = 0.893, which was a good level. The Social Relationship Scale was developed by Weiss (30) and adapted by Kim (31) to be suitable for South Korea. For social relationship (6.121), one factor and 10 questions were derived. The reliability of this is *α* = 0.926, which is a good level.

The Sports Environment Scale was developed by Stahl et al. (32) and modified and supplemented by Yang et al. (33) to make it suitable for South Korea. From sports environment (3.669), one factor and six questions were derived, and their reliability was *α* = 0.871, which was found to be reliable.

The Age-Friendly Environment Scale was based on the guidelines for age-friendly cities presented by the WHO (34) and used the scale constructed by Kim (35), Jung and Park (36), and Jung and Jung (37). A total of 10 questions of three factors were derived, including five items in sociocultural environment (3.381), three items in physical environment (2.054), and two items in service environment (1.747). These were found to be reliable in the sociocultural environment (*α* = 0.882), physical environment (*α* = 0.733), and service environment (*α* = 0.855). The Fear of Death Scale was based on the FODS (Fear of Death Scale) of Collett-Lester (38) and the scale of Park (39), which were modified and supplemented to be suitable for South Korea. For fear of own death (2.147), three items were derived; for fear of the own dying (1.589), two items were derived. A total of five items of two factors were derived, and the reliability of these items was found to be reliable as fear of own death (*α* = 0.808) and fear of the own dying (*α* = 0.716). The Collectivism Scale, initially developed by Triandis and Gelfand (40), was subsequently adapted to the South Korean context through modifications and supplements conducted by Sun (41).

### 2.3 Research methods

We analyzed the results using statistical methods. Software (SPSS WIN 24.0) was used for frequency, factor, reliability, t-test, correlation, and discriminant analyses. We conducted a frequency analysis to evaluate participants’ characteristics. Subsequently, factor and reliability analyses was conducted to confirm whether the variables were appropriate for the study, and a t-test, correlation analysis, and discriminant analysis were conducted to determine the SARC-F group discrimination according to the variables.

## 3 Result

### 3.1 Verification of differences among SARC-F groups by ecological system factors

A t-test was conducted to analyze the differences in the ecological systems (ego integrity, goal content for exercise, social support, social relationships, media availability, sports environment, age-friendly environment, collectivism, and the fear of death scale) among the selected groups. Among the six sub-factors of ego integrity and goal content for exercise, there was a significant difference between attitude toward life (*t* = 8.997, *p* < 0.001), wise life (*t* = 8.396, *p* < 0.001), social affiliation (*t* = 2.042, *p* < 0.05), and health management (*t* = 9.699, *p* < 0.001). In all four subfactors, those with a SARC-F score of three or less were higher than those with a score of four or higher. There were statistically significant differences in social support (*t* = 4.718, *p* < 0.001), social relationships (*t* = 2.176, *p* < 0.05), and media availability (*t* = 3.614, *p* < 0.001) belonging to the microsystem. In all three factors, the group with a SARC-F score of three or less was higher than that with a score of four or higher. There was a statistically significant difference in the sports environment (*t* = 6.084, *p* < 0.001) belonging to the exosystem; the group with a SARC-F score of three or less was higher than that with a score of four or higher. There was a significant difference between the physical environment (*t* = 7.520, *p* < 0.001), service environment (*t* = 3.356, *p* < 0.01), collectivism (*t* = 4.142, *p* < 0.001), fear of the own dying (*t* = 4.244, *p* < 0.001), and fear of own death (*t* = 8.302, *p* < 0.001) of the macrosystem. The group with a SARC-F score of three or less was higher than that with a score of four or higher. The results of the examination of the differences between the screening groups for sarcopenia according to the ecological system factors are shown in Table 3.

**Table 3:** Result of t-test between ecological system factor and SARC-F group.

Variable	Sub-factor	SARC-F	M	S.D	*t*		Sig.
Ego integrity	Attitude toward life	1	3.953	0.739	8.997	***	0.000
		2	3.360	0.584			
	Wise life	1	3.854	0.566	8.396	***	0.000
		2	3.442	0.430			
Goal content for exercise	Social recognition	1	2.610	0.905	−1.545		0.124
		2	2.750	0.830			
	Social affiliation	1	3.615	0.729	2.042	*	0.043
		2	3.493	0.514			
	Health management	1	4.252	0.550	9.699	***	0.000
		2	3.733	0.487			
	Skill development	1	2.899	0.914	−0.749		0.455
		2	2.962	0.748			
Social support	Social support	1	3.766	0.672	4.718	***	0.000
		2	3.476	0.550			
Social relationship	Social relationship	1	3.549	0.699	2.176	*	0.031
		2	3.407	0.589			
Media availability	Media availability	1	3.821	1.026	3.614	***	0.000
		2	3.308	1.373			
Sports environment	Sports environment	1	3.894	0.646	6.084	***	0.000
		2	3.538	0.521			
Age-friendly environment	Social environment	1	3.007	0.723	0.291		0.771
		2	2.987	0.623			
	Physical environment	1	4.122	0.591	7.520	***	0.000
		2	3.721	0.474			
	Health and community support service	1	3.665	0.874	3.356	**	0.001
		2	3.428	0.605			
Collectivism	collectivism	1	3.949	0.491	4.142	***	0.000
		2	3.757	0.417			
Fear of death scale	Own dying	1	2.895	0.866	4.244	***	0.000
		2	2.526	0.797			
	Own death	1	3.599	0.830	8.302	***	0.000
		2	2.962	0.689			

*SARC-F Group 1 = (score ≤ 3), Group 2 = (score ≥ 4). *p < 0.05; **p < 0.01; ***p < 0.001.

### 3.2 Results of correlation analysis between sub-factors of ecological system

Table 4 shows the results of the correlation analysis between the sub-factors of the ecological system. Specifically, ego integrity (attitude toward life, wise life), goal content for exercise (social recognition, social affiliation, health management, skill development), social support, social relationship, media availability, sports environment, age-friendly environment (social environment, physical environment, health and community support service), collectivism, and fear of death (own dying, own death) were correlated.

**Table 4:** Results of correlation analysis between ecological system factors.

	1	2	3	4	5	6	7	8	9	10	11	12	13	14	15	16
1	1.000															
2	0.515**	1.000														
3	−0.053	0.046	1.000													
4	0.198**	0.221**	0.289**	1.000												
5	0.339**	0.357**	0.039	0.386**	1.000											
6	0.015	0.080*	0.557**	0.347**	0.110**	1.000										
7	0.212**	0.299**	0.061	0.264**	0.283**	0.107**	1.000									
8	0.363**	0.377**	0.125**	0.439**	0.242**	0.226**	0.215**	1.000								
9	0.029	0.208**	0.024	0.041	0.104**	0.082*	0.186**	0.032	1.000							
10	0.304**	0.338**	0.014	0.323**	0.357**	0.107**	0.295**	0.350**	0.142**	1.000						
11	0.132**	0.291**	0.158**	0.235**	0.104**	0.185**	0.206**	0.238**	0.149**	0.443**	1.000					
12	0.352**	0.331**	0.006	0.263**	0.345**	0.036	0.229**	0.261**	0.091*	0.505**	0.343**	1.000				
13	0.243**	0.313**	0.074	0.226**	0.224**	0.109**	0.156**	0.237**	0.093*	0.428**	0.441**	0.432**	1.000			
14	0.364**	0.456**	0.020	0.308**	0.250**	0.097*	0.265**	0.409**	0.061	0.383**	0.204**	0.344**	0.242**	1.000		
15	0.303**	0.208**	−0.042	−0.018	0.078	0.010	0.064	0.001	0.065	0.027	0.087*	0.049	0.097*	−0.004	1.000	
16	0.548**	0.318**	−0.142**	0.054	0.235**	−0.107**	0.112**	0.138**	0.047	0.214**	0.077	0.225**	0.196**	0.154**	0.359**	1.000

1 = attitude toward life, 2 = wise life, 3 = social recognition, 4 = social affiliation, 5 = health management, 6 = skill development, 7 = social support, 8 = social relationship, 9 = media availability, 10 = sports environment, 11 = social environment, 12 = physical environment, 13 = health and community support service, 14 = collectivism, 15 = fear of the own dying, 16 = fear of own death.

### 3.3 Discriminant analysis between SARC-F groups by ecological system factors

Discriminant analysis was used to assess whether the SARC-F score group could be differentiated based on ecological system factors. This analysis aimed to determine which ecological system factors significantly contributed to the classification of individuals into different SARC-F score groups, providing valuable insights into the role of these factors in sarcopenia discrimination among the studied population. Table 5 presents the results.

**Table 5:** Discriminant analysis by SARC-F group by ecological system factors.

Variable	Function 1	
	Standardized canonical discriminant function coefficients	Structure matrix
Attitude toward life	0.185	0.582
Wise life	0.199	0.529
Social affiliation	−0.156	0.123
Health management	0.501	0.676
Social support	0.073	0.312
Social relationship	−0.124	0.147
Media availability	0.285	0.330
Sports environment	0.157	0.399
Physical environment	0.296	0.491
Health and community support service	−0.093	0.200
Collectivism	−0.008	0.281
Fear of the own dying	0.128	0.303
Fear of own death	0.265	0.554
Eigen value	0.285	
Explanatory variables	100	
Canonical Correlation	0.471	
Wilk’s λ	0.778^***^	

The discriminant analysis produced a single significant discriminant function (Wilks’s λ = 0.778, *p* < 0.001). This function comprises attitudes toward life, wise life, social affiliation, health management, social support, social relationships, media availability, sports environment, physical environment, service environment, collectivism, fear of the own dying, and fear of own death.

The structure matrix refers to the semantic relationship with the discriminant function health management (0.676), attitude toward life (0.582), fear of own death (0.554), wise life (0.529), physical environment (0.491), sports environment (0.399), media availability (0.330), social support (0.312), fear of own dying (0.303), collectivism (0.281), health and community support service (0.200), social relationship (0.147), and social affiliation (0.123). Standardized canonical discrimination parameters mean discriminant predictive power, in the order of health management (0.501), physical environment (0.296), media availability (0.285), fear of own death (0.265), wise life (0.199), attitude toward life (0.185), sports environment (0.157), fear of own dying (0.128), social support (0.073), collectivism (−0.008), health and community support service (−0.093), social relationship (−0.124), and social affiliation (−0.156).

The covariates between predicted variables and discriminant functions were health management (45.7%), attitude toward life (28.0%), fear of own death (30.7%), wise life (28.0%), physical environment (24.1%), sports environment (15.9%), media availability (10.9%), social support (9.7%), fear of the own dying (9.2%), collectivism (7.9%), health and community support service (4.0%), social relationship (2.2%), and social affiliation (1.5%). This implies that each factor has a common variance with a high specific gravity in the extracted discriminant function.

Table 6 shows the average discriminant function (centroid) of groups with three points or less and groups with four points or more based on SARC-F. According to the comparative analysis of the average value of the calculated discriminant function, the average value of the discriminant function in the first group with a SARC-F score of three or less was 0.240, and the average value of the second group with a SARC-F score of four or more was-1.185. This indicated that the two groups were distinguished using the extracted discriminant function.

**Table 6:** Average of discriminant functions by SARC-F group.

Division	Function
	1
Score ≤ 3	0.240
Score ≥ 4	−1.185

**Figure 1:**
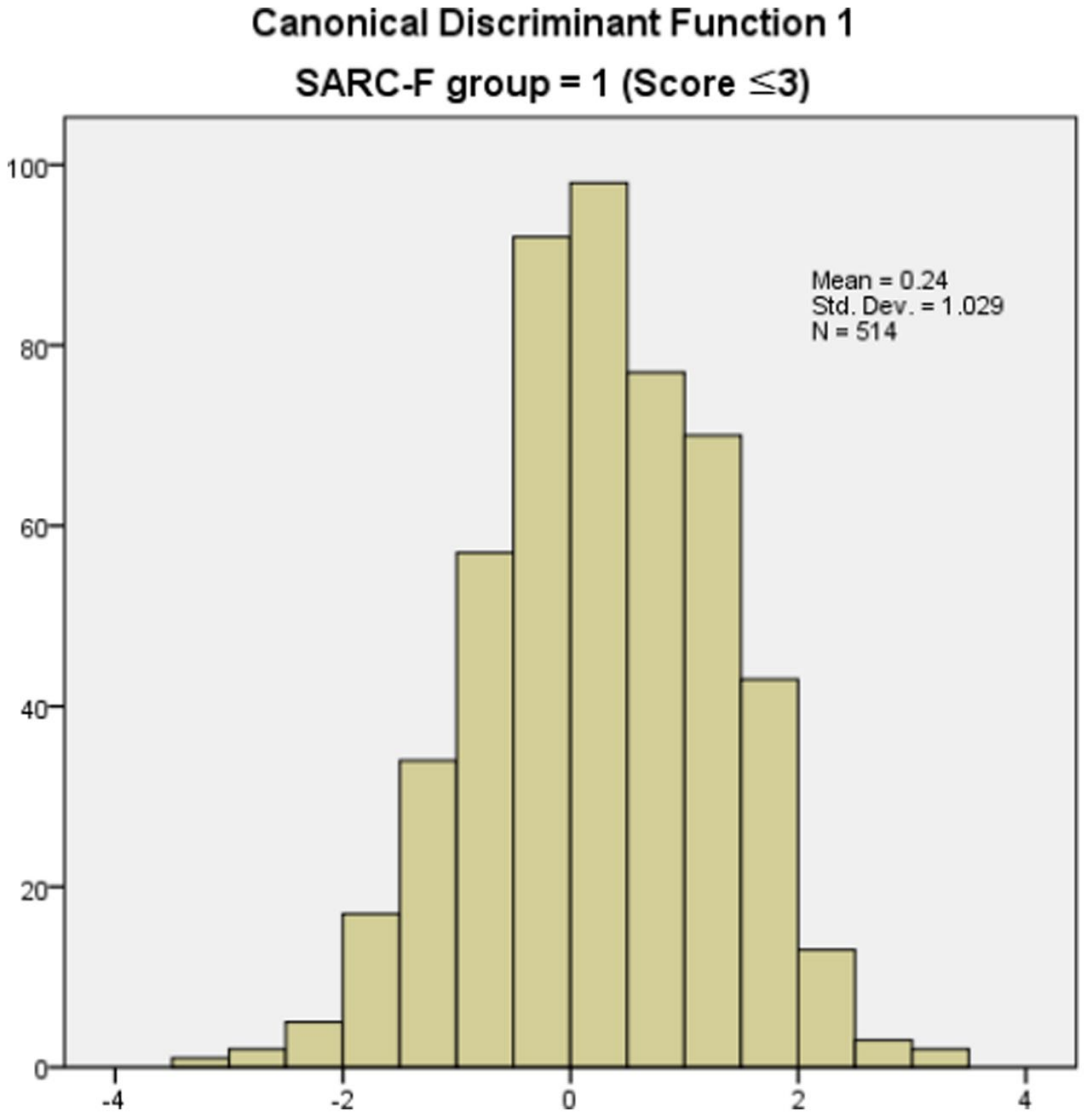
Canonical discriminant function 1 (SARC-F score ≤ 3).

**Figure 2:**
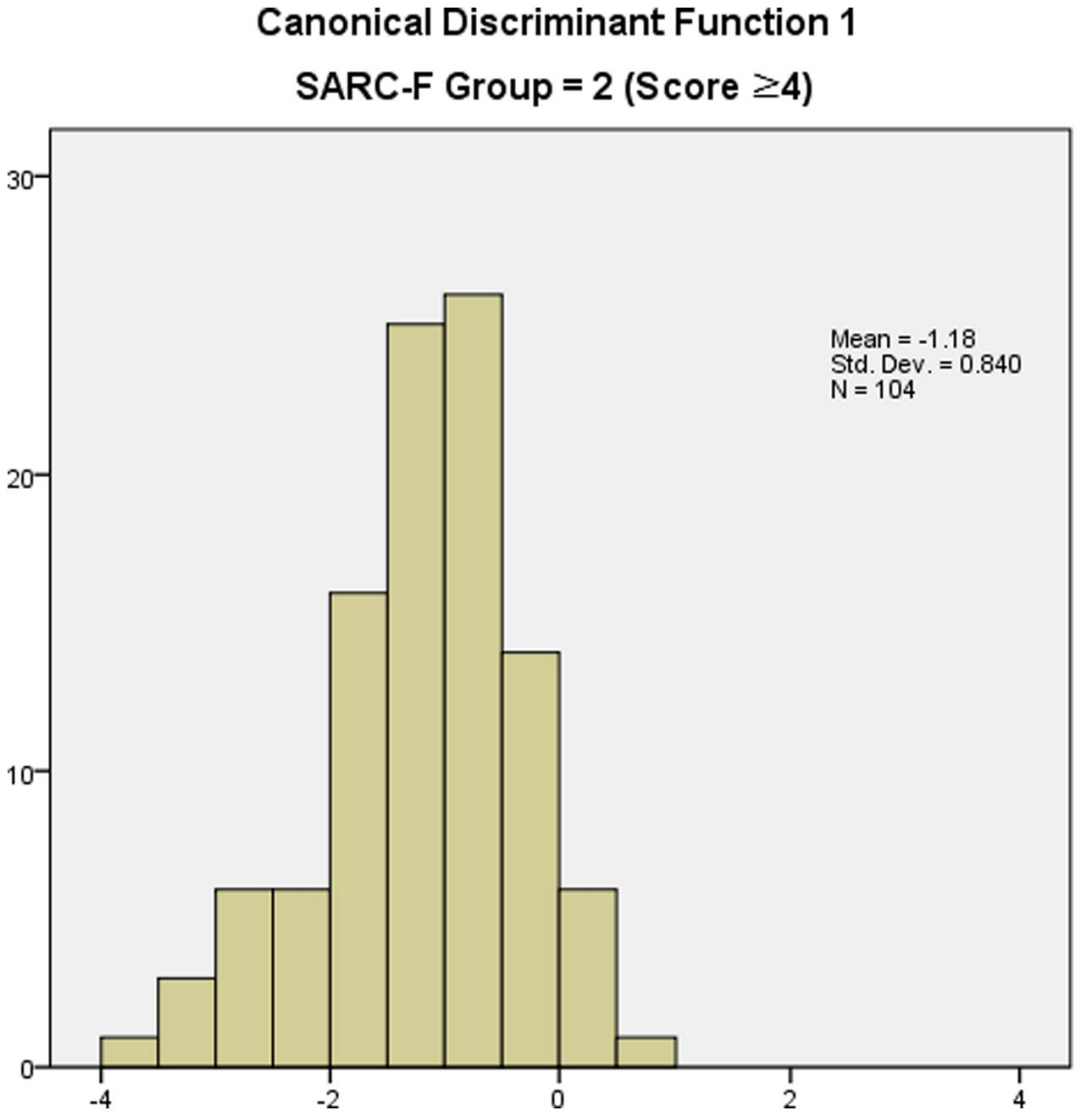
Canonical discriminant function 1 (SARC-F score ≥ 4).

The classification results based on the discriminant function coefficient is listed in Table 7. Specifically, in the case of the normal group according to the SARC-F, 493 people (95.9% of the total 514 people) were correctly classified, and 21 people (4.1%) were incorrectly classified. In the case of the risk group according to SARC-F, 29 people (27.9% of the total 104 people) were classified as normal, and 75 people (72.1%) were wrongly classified. Of the 618 participants, 522 were correctly classified according to the group type, and this discriminant function showed an accuracy of 83.5%. Figures 1, 2 show the canonical discriminant function by group.

**Table 7:** Classification results of SARC-F group by discriminant function coefficient.

Division	Predicted Group		*n*
	Score ≤ 3	Score ≥ 4	
Score ≤ 3	493 (95.9%)	21 (4.1%)	514
Score ≥ 4	75 (72.1%)	29 (27.9%)	104
Total	568	50	618

83.5% of original grouped cases correctly classified.

## 4 Discussion

This study aimed to identify the environmental factors that affect the incidence of sarcopenia in the aging Korean population through discriminant analysis. The implications of these results are discussed below.

First, the factors affecting discrimination of the sarcopenia group according to the SARC-F were health management, attitude toward life, fear of own death, wise life, physical environment, sports environment, media availability, social support, fear of the own dying, collectivism, health and community support services, social relationships, and social affiliation. If these sub-factors are rearranged into main factors, they will be arranged in the following order: goal content for exercise, ego integrity, fear of own death, age-friendly environment, sports environment, and media availability. If rearranged into Bronfenbrenner’s ecological system, the individual, macrosystem, exosystem, and microsystem can be seen in that order. Bronfenbrenner systematically classified ecological factors that affect human development and life and stated that these systems are influenced by interactions, not individuals (22). In other words, each ecological factor can be considered a factor influencing each other, not an individual variable. The results of this study show that the most important factors in determining the sarcopenia risk group of the older population in South Korea are individual-related factors. As the value of life of an older person becomes more important, many countries worldwide are improving their institutional and environmental aspects (42, 43). The government of the Republic of Korea is also promoting various policies to increase the number of institutions and medical facilities for the older adults and improve their residential environment. These positive effects are confirmed through institutional and policy improvements (11, 44, 45). Nevertheless, the results of this study confirm that improvements from a new perspective are necessary. For the older individuals to prevent or alleviate diseases such as sarcopenia, visible and invisible environmental improvements are also necessary. Nonetheless, simultaneously, the older individuals must be aware of themselves. Health management, attitudes toward life, and wise life, which have a primary impact on the determination of sarcopenia groups, are related to individuals. In other words, it is a characteristic of the human personality. Personality development is intricately influenced by environmental factors and consequently exerts a profound impact on physical and mental well-being (46). Therefore, it is important to educate the older population to recognize the importance of health and have a positive attitude toward life (47, 48). To this end, the government, local governments, and related organizations should provide programs for the older adults to act as members of society so that they can change their negative perceptions of aging. Additionally, due to the negative image of aging in society, many pre-older adults and young adults suffer from aging anxiety (49, 50). The education system should be changed so that the older adults and all other age groups can improve their perception of aging. In this regard, there is a lot of education in South Korea. As the older generation is no longer a socially marginalized class with poor information utilization ability, it is believed that realistic, specialized education, not blind education, should be conducted to improve awareness.

Second, the factors that had little influence on discriminating the sarcopenia group according to the SARC-F were collectivism, health and community support services, social relationships, and social affiliation. Reorganizing this into Bronfenbrenner’s ecological system appeared in the order of macro system, micro system, and organism, and most of the factors were related to interactions with others. This generally targets peers, workplaces, and neighbors for social activities and groups. However, it is believed that this is because the number of older individuals in South Korea interacting with others has declined after retirement. In other words, as personal characteristics were important in determining the sarcopenia group according to the SARC-F, interaction with others, for the older adults, is likely to be a reduced relationship, such as with family and close neighbors, rather than a general existence. As the relationship reduces, the older adults try to spend more time with their families and neighbors (51, 52). Currently, South Korea is experiencing rapid demographic changes due to a decrease in fertility rates, a decrease in marriage rates, and an increase in life expectancy. The country is on the verge of entering a super-aged society (2), and is living in an era centered around the individual, surpassing the era of nuclear families. In other words, the composition of society is changing, as is the composition of the furniture that constitutes it. At this point, it is obvious that the objects with which the olders interact will gradually be reduced. As the object of interaction has an important meaning for the olders, it is necessary to prepare various systems for the olders to form and maintain new relationships with. The interaction of the olders promotes social participation, and lifestyle habits are effective in treating and preventing major diseases in the olders (4, 53–55).

Third, based on the classification of the SARC-F group by the discriminant function coefficient, the groups with three points or less was classified with 95.9% accuracy, the groups with four points or more was classified with 27.9% accuracy, and the overall classification accuracy was 83.5%. The SARC-F is a key indicator recommended for case findings prior to the diagnosis of sarcopenia. These findings imply that the SARC-F has sufficient explanatory power to be used as basic data for screening sarcopenia. However, more diverse variables must be used to obtain accurate measurements.

The SARC-F consists of five questions that assess various aspects related to muscle strength and physical function in older individuals. The questions are: (a) How much difficulty do you have in lifting and carrying 10 lb.?; (b) How much difficulty do you have walking across a room?; (c) How much difficulty do you have transferring yourself from a chair or bed?; (d) How much difficulty do you have climbing a flight of 10 stairs?; (e) How many times have you fallen in the past year? Each question focuses on different aspects of physical ability and mobility. There is an opinion that sensitivity to questions constructed in this manner is not high. However, it was still used during the case-finding stage. Meaningful indicators should be created by increasing the question diversity. Therefore, at the same time as asking about physical abilities through SARC-F, through this study, it is expected that more accurate indicators will be constructed by questioning the ecological system factors that showed significant differences between groups.

This study had several limitations. First, in this study, the group with a SARC-F score of four or more was about 16.8% of the total, which is a large difference from the group with three or fewer points. Although this shows a similar ratio to the results of previous studies, it is necessary to secure and analyze the number of groups with more than four points for accurate results. Second, the SARC-F question is the first indicator that plays the role of case-finding in the process of screening for sarcopenia. Therefore, it is necessary to identify more accurate factors through an influence test using sarcopenia-related data from actual measurements.

## 5 Conclusion

In this study, discriminant analysis was conducted on the ecological system factors affecting sarcopenia screening items in the older population in South Korea. Among the data collected through online and offline surveys, 618 data points were used for analysis. A t-test and discriminant analysis were conducted to identify the differences between the SARC-F groups, a sarcopenia screening question, and the ecological system factors that influence it. The main results are as follows:

First, the ecological system that affects the discrimination of sarcopenia groups according to SARC-F was in the order of individual, macrosystem, exosystem, and microsystem. These findings suggest the necessity for a paradigm shift, particularly in the current emphasis on institutional and environmental factors to enhance the quality of life in old age.

Second, the ecological system, which exerted minimal influence on discriminating the sarcopenia group according to SARC-F, was primarily associated with interpersonal interactions. However, it is a clear task to complement the declining old age human network after retirement. This necessitates not only the supplementation of environments and facilities but also the establishment of diverse systems to enable the older generation to forge and sustain new relationships. Ultimately, such initiatives will prove effective in both treating and preventing diseases among older adults.

Third, the potential for enhancing current indicators, which solely assess physical abilities to diagnose sarcopenia, has been identified. A more precise screening index for sarcopenia could be developed by concurrently surveying both SARC-F and ecological system factors.

These results imply that in addition to confirming physical function through the SARC-F, ecological system factors can be used as significant indicators in the process of identifying sarcopenia in the olders adults in South Korea. As diseases such as sarcopenia affect the quality of life of the older adults, it is important not only to resolve the problem quickly, but prevent or minimize it from occurring. Therefore, the results of these studies can be used as basic data to help various age groups, such as the older adults in South Korea and prospective older to understand sarcopenia and solve its related problems.

## Data availability statement

The original contributions presented in the study are included in the article/supplementary materials, further inquiries can be directed to the corresponding author.

## Ethics statement

The studies involving humans were approved by Institutional Review Board of the Gachon University (No. 2023–020, 22 Mar 2023). The studies were conducted in accordance with the local legislation and institutional requirements. Written informed consent for participation in this study was provided by the participants’ legal guardians/next of kin.

## Author contributions

YR: Writing – original draft, Validation, Software, Methodology, Investigation, Formal analysis, Conceptualization. IC: Writing – review & editing, Validation, Supervision, Methodology, Formal analysis, Data curation. JK: Writing – review & editing, Validation, Supervision, Resources, Project administration, Funding acquisition, Formal analysis, Data curation.
